# Does Pulsed Magnetic Field Therapy Influence Nerve Regeneration in the Median Nerve Model of the Rat?

**DOI:** 10.1155/2014/401760

**Published:** 2014-07-21

**Authors:** Benedicta E. Beck-Broichsitter, Androniki Lamia, Stefano Geuna, Federica Fregnan, Ralf Smeets, Stephan T. Becker, Nektarios Sinis

**Affiliations:** ^1^Department of Oral and Maxillofacial Surgery, University Medical Center Hamburg-Eppendorf, Martinistraße 52, Campus Forschung Gebäude N27, 20246 Hamburg, Germany; ^2^Clinic for Plastic Surgery with Hand and Reconstructive Microsurgery, St. Marien Hospital, Gallwitzallee 123-143, 12249 Berlin, Germany; ^3^Department of Clinical and Biological Sciences, University of Turin, Regione Gonzole 10, 10043 Orbassano, Torino, Italy; ^4^Department of Oral and Maxillofacial Surgery, Schleswig-Holstein University Hospital, Arnold-Heller-Straße 3, Haus 26, 24105 Kiel, Schleswig-Holstein, Germany

## Abstract

The aim of this study was to evaluate the impact of pulsed magnetic field therapy on peripheral nerve regeneration after median nerve injury and primary coaptation in the rat. Both median nerves were surgically exposed and denervated in 24 female Wistar rats. A microsurgical coaptation was performed on the right side, whereas on the left side a spontaneous healing was prevented. The study group underwent a daily pulsed magnetic field therapy; the other group served as a control group. The grasping force was recorded 2 weeks after the surgical intervention for a period of 12 weeks. The right median nerve was excised and histologically examined. The histomorphometric data and the functional assessments were analyzed by *t*-test statistics and one-way ANOVA. One-way ANOVA indicated a statistically significant influence of group affiliation and grasping force (*P* = 0.0078). Grasping strength was higher on a significant level in the experimental group compared to the control group permanently from the 9th week to the end of the study. *T*-test statistics revealed a significantly higher weight of the flexor digitorum sublimis muscle (*P* = 0.0385) in the experimental group. The histological evaluation did not reveal any statistically significant differences concerning the histomorphometric parameters. Our results suggest that the pulsed magnetic field therapy has a positive influence on the functional aspects of neural regeneration. More studies are needed to precisely evaluate and optimize the intensity and duration of the application.

## 1. Introduction

Injuries of the peripheral nerve system still remain a great challenge in reconstructive surgery [1]. The outcome of recovering nerve function remains highly dependent on the period of time between injury and nerve repair in order to prevent irreversible muscular atrophy due to denervation of the target muscles [2–4]. For decades now, operative techniques have evolved to restore the nerve continuity from primary coaptation to autologous nerve grafts [5, 6] in order to achieve a reconstruction without any tension. If these methods are not applicable and nerve continuity cannot be restored otherwise [7–9], nerve transfer techniques are performed. Here, scientific approaches to create artificial nerve structures to improve the outcome and replace the autologous nerve grafting procedures in order to avoid the accompanying comorbidities have to be mentioned as well [6, 10, 11]. But even if the nerve continuity is restored immediately by means of a primary nerve coaptation or even with complex nerve transfers, depending always on the pattern of nerve injury, the patient oftentimes does not regain full complete nerve function [12].

Due to the existing limitations of surgical repair, neural regeneration may be additionally improved, supported, or influenced by perioperative management or applications, for example, physiotherapy, electrophysiology, or pulsed magnetic field therapy. A positive influence on osteoblast activity and bone healing processes as well as on neural regeneration has already been shown in the past [13–16].

In this study, we aim to evaluate the valence of electromagnetic stimulation in the neural regenerative process of the median nerve after neurotomy and primary coaptation in a rat model.

## 2. Materials and Methods

After final approval of the experimental study protocol according to the German and European Union guidelines (Permit-Nr. V312-72241.121-14 (124-10/11)) a total of 24 3-month-old female Wistar rats (Charles River Laboratories International, Wilmington, USA) with an average weight of 180 to 200 grams were used. Food and water were provided ad libitum in special housings for 4 animals in each cage. The laboratory environment secured a room temperature of 20° Celsius with a relative humidity of 55 ± 10% and a night-day-rhythm of 12 hours each.

### 2.1. Experimental Design

General anaesthesia with Sevoflurane (Sevorane, Abbott, Baar, Switzerland) was applied for every surgical procedure with additional use of a Zeiss surgical microscope (Carl Zeiss AG, Jena, Germany) for microsurgical techniques. The vaporisation of Sevoflurane was guided through specialized devices (Vapor, Drägerwerk, Lübeck, Germany).

Before the surgical procedure, the forelegs were shaved and afterwards disinfected (Kodan, Schülke&Mayr, Norderstedt, Germany). In a state of sufficiently deep anesthesia, the median nerve was carefully exposed from the axilla to the cubital fossa after skin incision. After neurotomy of the right median nerve, both nerve stumps were primarily coapted applying two microepineural single knot sutures (11-0 Nylon, Resolon, Ethicon, Norderstedt, Germany). The left median nerve was dissected and, afterwards, 20 millimeters were excised in order to prevent a spontaneous healing. The wounds were closed with resorbable suturing materials (Vicryl, Ethicon, Norderstedt, Germany).

**Figure 1:**
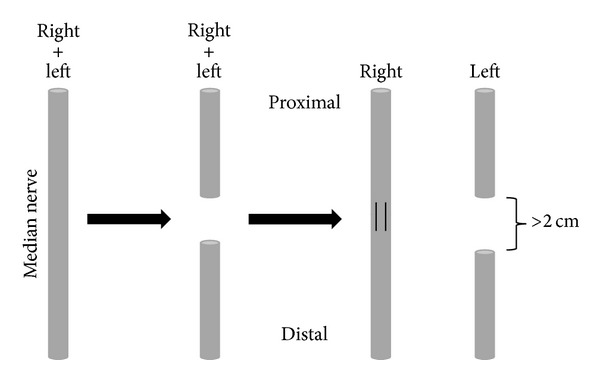
Scheme of surgical protocol. Both median nerves were exposed and denervated. The right median nerve was mircrosurgically coapted. Spontaneous healing on the left side was prevented by excision.

**Figure 2:**
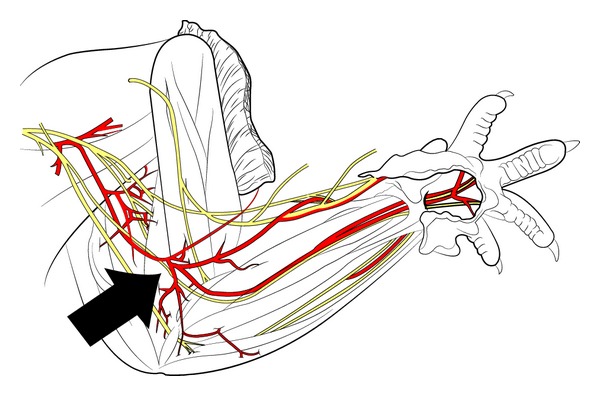
The drawing displays the rat's anatomy of the upper extremity (adopted from Greene 1935) [17]. The arrow points in the area of denervation.

Each of the 12 animals was then randomized in a control and a study group. The surgical study setup schematically displayed in Figures 1 and 2 provides an overview of the anatomy.

Tramadol (Tramal, Grünenthal, Aachen, Germany; 0.002 mg/g body weight) was applied after surgery for additional 5 days. The study group received daily a pulsed magnetic therapy provided by a magnetic matrace (Bemer 3000, Bemer Int., Triesen, Switzerland) since the first day after the operation. The stimulus was applied for 12 minutes daily using a 35 Microtesla matrace, 33 Hz, every 150 milliseconds. Two weeks after the surgery, functional assessments were performed over an additional 12-week period.

At the end of the study the right median nerve was excised after sacrificing the animals by CO_2_-insufflation. The wet weight of the flexor digitorum sublimis muscle was then determined. The analysis aimed to determine weight development compared between both groups as a reference for the degree of successful reconstruction of the innervating median nerve under the additional influence of a pulsed magnetic field therapy.

### 2.2. Functional Assessment

Two weeks after the nerve reconstruction, grasping tests, first described by Bertelli and Mira [18], were performed to follow the functional neural regeneration after the median nerve injury. This test should objectively describe the peripheral nerve regeneration in the rat after a median nerve injury: The flexor digitorum sublimis muscle is solely innervated by the median nerve and leads to finger flexion. Here, a wire grid (8 × 14 cm) is fixed on an electric balance. Each animal is gently lifted by the tail in order to grasp for the wire grid. After three attempts, the maximal grasping force is recorded on the electronic balance as soon as the animals lose their grip. The grasping of digits without flexion of the elbow or wrist was evaluated. Only one and always the same person performed the grasping tests in a blinded technique in order to avoid an observer bias. Body weights were recorded previous to the grasping test weekly.

### 2.3. Histological Evaluation

After sacrificing the animals, the median nerve cables on the right side were excised and fixed in 2.5% glutaraldehyde, washed in Sorensen phosphate buffer 0.1 M (pH 7.4) with 1.5% sacarose, and post-fixed in 2% osmium tetroxide for 2 hours. The samples were dehydrated by means of ethanol and cleared in propylene oxide afterwards. The samples were then covered in a Glauert's embedding mixture of resins, consisting of Araldite M and Araldite Harter in equal parts, HY 964 (Merck, Darmstadt, Germany), containing 0.5% of the plasticizer dibutyl phthalate, and 1-2% of the accelerator 964, DY 064 (Merck).

### 2.4. Morphometric Assessment

The nerve samples were stained with toluidine blue after being cut from distally in 2.5 millimeter cross-sections with an ultramicrotome (Ultracut, Leica, Wetzlar, Germany). Morphometric analysis was conducted on 6 animals for each experimental condition. The sections for the morphometrical analysis were randomly chosen in the last third of the nerve cable and the analysis was carried out using a DM4000B microscope with a DFC320 digital camera and an IM50 image manager system (Leica Microsystems, Wetzlar, Germany).

A final 6600-fold magnification secured an accurate identification of myelinated nerve fibers. At first, a randomly selected nerve specimen and its total cross-sectional area was evaluated at a lower magnification. Following a randomization protocol, a sampling of the nerve fibers was carried out. According to a previous publication, a bias due to the “edge effect” was avoided by adoption of a two-dimensional dissector procedure [19]. A sample of myelinated nerve fibers in two-dimensional dissector probes was also used to select an unbiased representative. For each fiber, fiber and axon surface were measured and the circle-fitting diameter of axon (*d*) and fiber (*D*) was calculated. These data were used to calculate myelin thickness [(*D* − *d*)/2], myelin thickness/axon diameter ratio [(*D* − *d*)/2*d*], and axon/fiber diameter ratio, the *g*-ratio (D/d).

### 2.5. Statistical Analysis

The development of the grasping force between the study and the control group during the study was analyzed for statistical significant differences using the one-way ANOVA. Here, group affiliation and time point of measurements during the observation period (12 weeks) were defined as influence factors. The animal was identified as a random factor. Parameters and measured values gathered from histomorphometric assessments as well as grasping force comparisons at different time points were further analyzed by unpaired two-sample *t*-test statistics accordingly. The level of significance was set to 0.05.

## 3. Results

### 3.1. Functional Assessment

In the beginning of the observation period, the mean grasping force was determined at 87.4 g (SD: ±34.8 g) in the experimental group and 64.3 g (SD: ±23.2 g) in the control group (*P* = 0.0683). Grasping strength increased over time and led to a statistically significant difference between the group receiving magnetic field therapy (238.1 g; SD ±88.9 g) compared to the control group 175.2 g (SD: ±40.6 g) (*P* = 0.0153).

The statistical analysis of grasping force development comparing both groups at every time point in the observation period indicated a statistically significant difference in favor of the experimental group starting from the 9th week on to the end of the study.  Table 1 provides an overview of group comparisons and *P* values, respectively.

**Figure 3:**
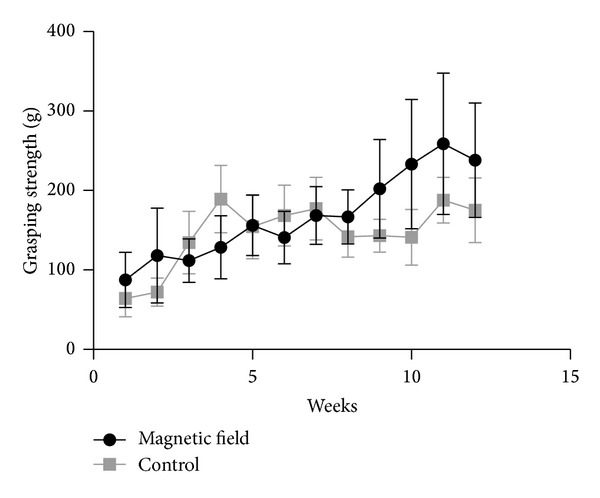
Development of grasping force during the study comparing the study group receiving a pulsed magnetic field therapy and the control group. Since the 7th measurement grasping strength in the group of magnetic field therapy increases on a higher level compared to the control group. Statistical analysis between both groups revealed statistically significant differences from the 9th measurement to the end of the observation period in favor for the experimental group.

The course of measurements is displayed in Figure 3. One-way ANOVA of grasping forces revealed a significant difference for grasping strength according to group affiliation (*P* = 0.0078).

**Figure 4:**
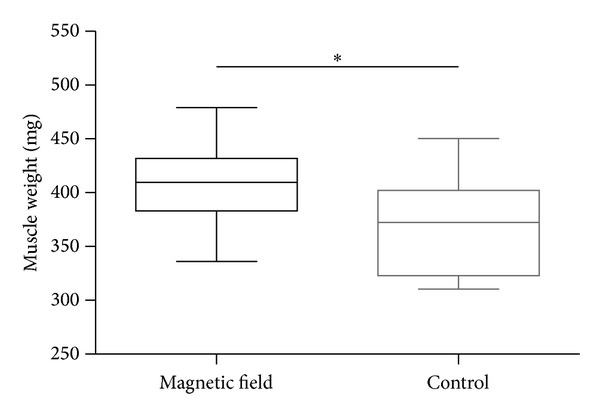
Comparison of the flexor digitorum sublimis muscle weight between study group and control group indicates a significantly higher weight in favor of the experimental group (*P* = 0.0385).

The comparison of muscle weight was also significantly higher in the study group (*P* = 0.0385; Figure 4). Mean wet muscle weight revealed 406.8 mg (SD: ±40.7 mg) in the group with the pulsed magnetic field therapy compared to 367.5 mg the control group (SD: ±46.5 mg).

### 3.2. Histomorphometric Assessment

Table 2 includes the total number of nerve fibers in the sample and their density related to the total area of the nerve fibers. The total number (mean 6517, SD: 3265) and surface area (mean 0.3916 mm^2^, SD: 0.2137 mm^2^) were higher in the group with the pulsed magnetic field treatment compared to the control group (mean 5292, SD: 2248; mean 0.2718 mm^2^, SD: 0.0707 mm^2^), whereas the density remained higher in the control group (21022 mm^−2^, SD: 10853 mm^−2^; pulsed magnetic field treatment: 17646 mm^−2^, SD: 7374 mm^−2^), but, statistically, there were no differences found on a significance level of 5%.

In Table 3, parameters characterizing the nerve fiber are summarized. The nerve fiber thickness remained apparently not different between both groups (pulsed magnetic field treatment: 3.65 *μ*m, SD: 0.21 *μ*m; control group: 3.62 *μ*m, SD: 0.35 *μ*m). The Myelin sheet revealed averagely higher values in the pulsed magnetic field treatment group (1.32 *μ*m, SD: 0.65 *μ*m) compared to control group (1.05 *μ*m, SD: 0.51 *μ*m) and so did the axon diameter (pulsed magnetic field treatment: 2.47 *μ*m, SD: 0.33 *μ*m; control group: 2.28 *μ*m, SD: 0.24 *μ*m). These differences were not statistically different.

The *g*-ratio (0.97, SD: 0.31) and* M/d*-ratio (0.93, SD: 0.56) brought higher values for the group with the pulsed magnetic field treatment compared to the control group (0.81, SD: 0.3; 0.68, SD: 0.49), whereas the* D/d* ratio revealed higher values in the control group (1.62; SD: 0.13) than in the group with pulsed magnetic field treatment (1.54, SD: 0.09). Statistically significant differences could not be found on a significance level of 5% for these indices.

## 4. Discussion

Besides the surgical challenge and technical feasibility to restore the continuity of injured peripheral nerves, the functional outcome is oftentimes not satisfying. A full nerve recovery remains achievable for approximately 10% of the patients [10, 20, 21]. Here, the positive influence of a pulsed magnetic field, already shown concerning healing processes of the bone [13, 14] and peripheral nerves [15, 16], might have a positive impact on the functional postoperative outcome. The success of the treatment may be evaluated when considering the functional and morhological as well as histological aspects as a whole.

The histomorphometric assessments that were performed in our study in order to evaluate the nerve coaptation site for histological signs of neural regeneration indicated that there was no statistically significant difference between pulsed magnetic field treatment and the control group on a histological as well as on a morphological level indicating no influence of pulsed magnetic field therapy on the morphological aspects investigated in the study for signs of the median nerve's regeneration. These findings were underpinned by the findings of another workgroup, who performed a study on 34 mice, which partially underwent a three week low-frequency pulsed magnetic field treatment after a sciatic crush lesion. Histologically, there was no difference between the study and the control group but the histomorphometric evaluation revealed even a negative effect of the pulsed magnetic field treatment expressed by decreased regeneration and increased oxidative stress signs. A difference in functional recovery could not be proven [22]. Another sciatic nerve injury model study in the rat indicated that, after a low-frequency pulsed magnetic field therapy for 38 days, the Wallerian degeneration as well as the electrophysiological assessments remained comparable between the group receiving the pulsed magnetic field treatment and control group [23]. In a rat model study of the facial nerve regeneration after transection and reapproximation without suturing, the animals were treated four hours a day for a total of eight weeks with pulsed magnetic fields. To evaluate the neural regeneration electroneurography, the eyelid force, whisker movements, and voluntarily facial movements were assessed in comparison to the preoperative state and in two-week intervals after the intervention. They found behaviorally and electrophysiological beneficial influences in the study groups, though their results were not able to provide a conclusive proof [24]. In another rat model, the influence of a pulsed magnetic field application to a sciatic nerve lesion was evaluated. The study group consisted of animals with a primary coaptation and those having received an autologous nerve graft. A pulsed magnetic field therapy was applied 6 hours daily over a four-week period. In the study group, a larger number of myelinated nerve fibers as well as an enzyme activity (acetyl cholinesterase) at the endplate were recorded [25]. An acceleration of myelin sheath regeneration was also discussed as a possible effect of a long-periodic pulsed magnetic field treatment in another sciatic nerve injury model in the rat [26]. Gunay and Mert assessed the influence of conduction characteristics of a regenerating peripheral nerve in a sciatic crush lesion model in the rat over a 15- and 38-day period. Here, abnormalities in signaling and aberrant ion channel functions were time-dependently restored by the pulsed magnetic field therapy [27]. Another in vitro study could show that neurite growth was influenced by the direction of the magnetic field applied by nanoparticles [28, 29].

The higher grasping forces recorded in the animal group receiving the pulsed magnet field therapy during our observation period could indicate an improvement of aberrant cell signalling as well as a direct influence of magnetic fields on direction of cellular growth. This furthermore corresponds to our findings that the flexor digitorum sublimis muscle's weight of the same animal group was significantly higher signifying a positive impact on the biochemical processes on the endplate, the cellular membrane, or the muscle itself. We could also show that grasping strength was significantly higher favoring the experimental group from the 9th week to the end of the study indicating a significantly faster functional recovery of strength after an initial healing period.

A study of Currier et al. demonstrated in a group of 17 patients after a reconstructive surgery of the anterior cruciate ligament that a combination of neuromuscular electrical stimulation and pulsed magnetic field therapy was superior in the prevention of a massive girth reduction of the knee extensor muscles in comparison to a neuromuscular electrical stimulation alone [30]. A direct influence of a pulsed magnetic field therapy on a muscular level was provided in a study with a diabetic rat model investigating differences of wound healing and observing and recording the anti-smooth muscle actin immunohistochemistry. From their findings, it could be hypothesized that pulsed magnetic field therapy could increase the myofibroblast population [31]. There might also be a positive influence on striped skeletal muscle cells as well but it is not described so far. In another rat model, the gastrocnemius muscle was similarly denervated and the effect of electric stimulation versus magnetic stimulation between the two study groups was further evaluated. Muscular weight was significantly higher in the magnetic field treatment group when compared to electrical stimulation group [32].

## 5. Conclusion

We may conclude that a pulsed magnetic field therapy can positively influence the functional regeneration after a median nerve injury and primary coaptation in the rat. This was displayed by a reduced muscular atrophy and a higher grasping force at the end of the study observation period. In order to optimize the effects of a pulsed magnetic field therapy and apply them in the clinical field, more studies are needed in order to evaluate the different application durations and different pulsed magnetic fields intensities.

## Figures and Tables

**Table 1:** Mean-values and standard deviations of grasping force during the observation period are compared for every single measurement. *P* value indicated statistically significant differences in favor of the experimental group in the 9th week to the end of the observation period.

Time point	Magnetic field		Control		*P* value
	Mean-value	Standard deviation	Mean-value	Standard deviation	
1	87.44	34.76	64.33	23.16	0.0683
2	118.33	59.86	72.28	17.72	**0.0180**
3	111.69	27.62	134.47	39.33	0.1149
4	128.36	39.94	189.14	42.41	**0.0015**
5	156.22	38.11	154.58	40.52	0.9196
6	140.81	33.07	168.39	38.33	0.0724
7	168.53	36.5	177.14	39.44	0.5844
8	166.89	33.96	141.81	25.71	0.0536
9	202.33	62.13	143.11	20.78	**0.0049**
10	233.31	81.34	141.03	35.24	**0.0016**
11	258.89	88.93	187.78	28.86	**0.0151**
12	238.06	72.17	175.17	40.58	**0.0153**

**Table 2:** When comparing histomorphometric parameters between both experimental and control group no statistically significant differences could be determined.

Group	Total Number		Density (fibers/mm^2^)		Area (mm^2^)	
	Mean	SD	Mean	SD	Mean	SD
Magnetic Field	6517	3265	17646	7374	0.3916	0.2137
Control	5291	2248	21022	10853	0.2718	0.0707
*P*-value	0.509		0.581		0.268	

**Table 3:** When comparing histomorphometric parameters between both experimental and control group no statistically significant differences could be determined.

Group	Axon Diameter		Nerve Fibre Diameter		Myelin Thickness	
	Mean	SD	Mean	SD	Mean	SD
Magnetic Field	2.47	0.33	3.65	0.21	1.32	0.65
Control	2.28	0.24	3.62	0.35	1.05	0.51
*P*-value	0.341		0.912		0.485	
